# Stereotaxic Exposure of the Central Nucleus of the Amygdala to Corticosterone Increases Colonic Permeability and Reduces Nerve-Mediated Active Ion Transport in Rats

**DOI:** 10.3389/fnins.2018.00543

**Published:** 2018-08-07

**Authors:** Priya Hattay, Dawn K. Prusator, Anthony C. Johnson, Beverley Greenwood-Van Meerveld

**Affiliations:** ^1^Oklahoma Center for Neurosciences and Department of Physiology, University of Oklahoma Health Sciences Center, Oklahoma City, OK, United States; ^2^Veterans Affairs Medical Center, Oklahoma City, OK, United States

**Keywords:** amygdala, corticosterone, permeability, stress, rat, colon

## Abstract

**Background:** Irritable bowel syndrome (IBS) is characterized by visceral pain and abnormal bowel habits that are worsened during stress. Evidence also suggests altered intestinal barrier function in IBS. Previously, we demonstrated that stereotaxic application of the stress hormone corticosterone (CORT) onto the central nucleus of the amygdala (CeA) induces colonic hyperalgesia and anxiety-like behavior in a rat model, however the effect on intestinal permeability and mucosal function remain to be evaluated.

**Methods:** Male Fischer 344 rats underwent bilateral stereotaxic implantation of CORT or inert cholesterol (CHOL)-containing micropellets (30 μg) onto the dorsal margin of the CeA. Seven days later, colonic tissue was isolated to assess tissue permeability in modified Ussing chambers via transepithelial electrical resistance (TEER) and macromolecular flux of horseradish peroxidase (HRP). Secretory responses to electrical field stimulation (EFS) of submucosal enteric nerves as well as activation with forskolin were used to assess movements of ions across the isolated colonic tissues. In a separate cohort, colonic histology, and mast cell infiltration was assessed.

**Key Results:** Compared to CHOL-implanted controls, we determined that exposing the CeA to elevated levels of CORT significantly increased macromolecular flux across the colonic epithelial layer without changing TEER. Nerve-mediated but not cAMP-mediated active transport was inhibited in response to elevated amygdala CORT. There were no histological changes or increases in mast cell infiltration within colonic tissue from CORT treated animals.

**Conclusion and Inferences:** These observations support a novel role for the CeA as a modulator of nerve-mediated colonic epithelial function.

## Introduction

Irritable bowel syndrome (IBS) is a common gastrointestinal (GI) disorder associated with chronic abdominal pain accompanied by a change in stool frequency or consistency, and a 2:1 female predominance (Chang, 2004; Drossman and Dumitrascu, 2006; Foxx-Orenstein, 2006; Defrees and Bailey, 2017). Although the exact pathophysiological mechanisms underlying IBS remain incompletely understood, patients with IBS often experience worsening of their symptoms during periods of stress and negative emotions (Whitehead et al., 1992; Pellissier and Bonaz, 2017). An investigation into the stress responsivity of IBS patients reveals a potential abnormality of negative feedback control of the hypothalamic-pituitary-adrenal (HPA) axis (Chang, 2004) with functional magnetic resonance imaging studies revealing differential activation and deactivation of brain regions associated with central stress circuitry including the anterior cingulate cortex, prefrontal cortex, and amygdala in patients with IBS (Berman et al., 2008; Tanaka et al., 2016; Icenhour et al., 2017). The amygdala is an important structure for regulating anxiety, with the central nucleus of the amygdala (CeA) facilitating the activation of the HPA axis and the autonomic nervous system response to stress.

Visceral hypersensitivity characterized by hyperalgesia and allodynia has been measured in patients with IBS (Verne et al., 2001; Fuentes and Christianson, 2016). The cause of visceral hypersensitivity is likely multifactorial, involving both central and peripheral factors (Zhou et al., 2009; Barbara et al., 2011; Greenwood-Van Meerveld et al., 2015). Central factors include stress and anxiety while peripheral factors such as an acute episode of infective gastroenteritis and changes in the gut microbiota may contribute to pain perception by amplification of sensory signaling in IBS (Greenwood-Van Meerveld et al., 2015; Greenwood-Van Meerveld and Johnson, 2017). From both human studies and animal models, evidence points toward interplay between visceral hypersensitivity in IBS, low levels of inflammation and increased intestinal permeability (Piche et al., 2009; Barbara et al., 2011; Camilleri et al., 2012; Martinez et al., 2012). Abnormalities in epithelial permeability enhance the possibility of luminal antigens and toxins crossing the epithelial barrier. In the presence of enhanced intestinal permeability there is an increase in the movement of inflammatory mediators as well as gut microbiota across the gut wall, leading to activation of the immune system (Bischoff et al., 2014). Another potential consequence of increased intestinal permeability is potential sensitization of myenteric and spinal afferent nerve terminals leading to central sensitization and visceral hypersensitivity. In support, evidence in IBS patients has shown that following an acute enteric infection, IBS patients exhibit increased intestinal permeability and long term visceral hypersensitivity (Zhou et al., 2009; Zhou and Verne, 2011; Beatty et al., 2014). Together, these findings support the notion that increases in intestinal permeability could pay a role in contributing to a chronic nociceptive drive from the GI tract to the spinal cord leading to central sensitization and a hypersensitive gut, resembling that which is seen in a subset of IBS patients. In support, IBS patients (IBS with diarrhea 40%; IBS with constipation 4%) showed increased intestinal permeability, and those IBS patients with intestinal hyper-permeability also had higher functional bowel disorder severity index (FBDSI) scores compared to those without altered permeability. Furthermore, the increase in the FBDSI scores correlated positively with increases in visceral and thermal hypersensitivity (Zhou and Verne, 2011).

The gut mucosal barrier can be classified as leaky (small intestine) or tight (colon), depending on its ability to transport fluid and electrolytes. Sustaining gut barrier function and mucosal permeability are tight junction (TJ) proteins, which connect adjacent epithelial cells and regulate the paracellular movement of substances across the small and large intestinal mucosa. This paracellular movement of gut contents between the epithelial cells is therefore dependent on the composition of the TJ proteins found between cells, which include various combinations of zonula occludens (ZOs), occludins, claudins, and junctional adhesion molecules. Additionally, a layer of mucus covering the mucosal epithelium represents an “unstirred” layer and barrier to movement of contents between intestinal epithelial cells (Camilleri et al., 2012). A growing body of evidence suggests that alterations in barrier function may be an important factor in the pathogenesis of IBS, and we recently demonstrated that in response to a chronic psychological stressor there are increases in permeability, mucosal mast cells, and expression of pro-inflammatory cytokines along with altered TJ protein expression within the rat colon (Hattay et al., 2017); however, due to the systemic nature of the chronic stressor, the role of specific central and/or peripheral factors could not be determined, although there is evidence that corticotropin-releasing hormone via its type 2 receptors can play a role (Ducarouge et al., 2013, 2017). The goal of the present study was to test the hypothesis that a centrally driven facilitation of the stress axis via corticosterone (CORT)-induced activation of the CeA would be sufficient to induce abnormalities in colonic barrier function and increase colonic permeability. To address this goal, we selected an experimental rat model in which we could directly and specifically expose the CeA to elevated levels of CORT, without peripheral factors induced by repeated chronic stress induced-activation of the HPA-axis. Our previous studies using this model demonstrated that stereotaxic implantation of the CORT onto the dorsal margin of the CeA induced persistent anxiety-like behavior, chronic visceral hypersensitivity and colonic dysmotility in rats (Greenwood-Van Meerveld et al., 2001; Myers and Greenwood-Van Meerveld, 2007; Venkova et al., 2010) through a mechanism involving a down regulation of glucocorticoid receptors and an increased in corticotropin-releasing factor expression in the CeA (Johnson and Greenwood-Van Meerveld, 2015; Johnson et al., 2015; Tran et al., 2015).

## Materials and methods

### Animals and experimental design

**Figure 1:**
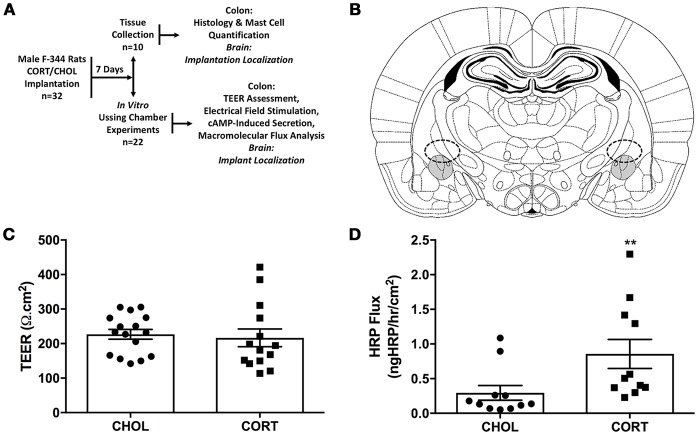
Elevated amygdala CORT significantly increases macromolecular flux across the colonic epithelium. **(A)** Experimental design: 32 male rats were randomly assigned to receive stereotaxic implantation of micropellets containing CORT or CHOL on the dorsal margin of the CeA. After a 7-day recovery, *n* = 10 rats (5 rats/treatment) were euthanized for tissue collection for evaluation of colonic histology, mast cell infiltration, and tight junction protein expression; *n* = 22 rats (11 rats/treatment) were euthanized and the colon was used for evaluation of mucosal permeability, macromolecular flux, neural mediated secretion, or receptor independent secretion. **(B)** Representative schematic of CORT or CHOL micropellet localization based upon the rat brain atlas of Paxinos and Watson (Paxinos and Watson, 2014). **(C)** Amygdala CORT did not affect colonic TEER (Mann-Whitney *U* = 88.5, *p* = 0.339) compared to CHOL treated controls (*n* = 5–6/group, 14–16 viable Ussing Chambers samples/treatment). **(D)** CORT implantation onto the amygdala significantly increases the flux of HRP across the colonic epithelium (Mann–Whitney *U* = 16, **p = 0.0024) (CHOL *n* = 5, 11 preparations; CORT *n* = 4, 11 preparations). Values are mean ± SEM.

Experiments were performed using 32 male Fischer 344 (F-344) rats, 250–300g (Charles River, Wilmington, MA). F-344 rats were chosen because they have been demonstrated to possess low basal levels of anxiety (Glowa and Hansen, 1994). Rats were housed in controlled conditions (30–70% humidity, 18–24°C) maintained on 412h:12h light- dark cycle (lights on at 6:00 a.m.), housed two per cage in standard rat cages (27.3 × 48.9 × 27.3 cm) with Sani-chip bedding material that were changed once weekly with standard rat food (Rat Diet 5053, LabDiet, St Louis, MO, USA) and water *ad libitum*. To reduce stress associated with shipping and the laboratory environment, rats were acclimated to the animal facility for at least 7 days followed by a second 7-day period of acclimatization to the experimental environment during surgical recovery. During this acclimatization period, rats were brought into the laboratory between the hours of 10:00 a.m. and 2:00 p.m., weighed, and handled by the investigator. All experiments were performed at the same time each day (between 10:00 a.m. and 2:00 P.M.). The animal protocol was approved by the Institutional Animal Care and Use Committee at the Oklahoma City VA Medical Center (1302-001) and the University of Oklahoma Health Sciences Center (13-028). After facility acclimation, all rats were randomized to treatment groups and underwent stereotaxic bilateral implantation of CORT or cholesterol (CHOL) control micropellets on the central nucleus of the amygdala (CeA) (Day 0). During recovery from the surgery all rats were single-housed. On day 7 after the surgery, rats were euthanized and colonic tissue was isolated for the measurement of transepithelial electrical resistance (TEER), macromolecular transport, active ion transport induced following nerve stimulation and/or the addition of forskolin. In a separate cohort of rats on day 7 after the stereotaxic bilateral implantation of CORT or CHOL micropellets on the CeA colonic tissue was isolated mast cell quantification and histology. Post mortem, the rat brain was isolated to confirm the accurate placement of the bilateral micropellet implant on the dorsal margin of the CeA (Figure 1A).

### Stereotaxic implantation of micropellets

Pharmacological manipulation utilized stereotaxic implantation of CORT or CHOL (MilliporeSigma, St. Louis, MO, USA) micropellets onto the dorsal surface of the CeA. Rats were anesthetized with a ketamine-xylazine cocktail administered intraperitoneally (100 mg/kg ketamine; 10 mg/kg xylazine) and body temperature was monitored during the surgical procedure and maintained at 37°C using a homeothermic heating blanket (Harvard, Ealing, UK). Animals were mounted in a stereotaxic frame and secured by both the ear and bite bars (Kopf, Tujunga, CA). A midline incision was made from the frontal region to the apex of the skull, and the epidermis and fascia were retracted. The stereotaxic frame was equipped with a digital coordinate system, which allowed for precise localization of the targeted brain region using skull landmarks for bregma and lamda. Following a midline incision, small holes were made in the skull at the coordinates 2.5 mm posterior to bregma and 4.2 mm to the right and the left of midline using the Paxinos-Watson stereotaxic atlas (Paxinos and Watson, 2014). Rats were then stereotaxically implanted over the dorsal margin of the CeA (bilaterally) with CORT (30 μg), shown previously to increase anxiety-like behavior and visceral pain (Shepard et al., 2000; Greenwood-Van Meerveld et al., 2001; Myers and Greenwood-Van Meerveld, 2007, 2010b), or inert CHOL (30 μg) by lowering a cannula 7.0 mm ventral to the surface of the dura. After the incision was sutured, antibiotic and analgesic cream was applied to the wound and buprenorphine (5 mg/kg, s.c.) was administered twice daily for post-operative analgesia. Surgeries were performed in a dedicated surgical area of the experimental laboratory between 10:00 a.m. and 2:00 p.m. Animals were then allowed seven days for recovery, during which time their behavior was observed to ensure that they were not in distress. The stereotaxic coordinates were previously shown to localize the CORT-contained micropellets to within a diffusible radius (750 μm) of the CeA (Shepard et al., 2003). Micropellet site localization performed post-mortem confirmed that all micropellets were correctly targeted to the dorsal surface of the CeA.

### Tissue isolation for ussing chamber studies

Within the main laboratory, starting at 10:00 a.m., after preparation of Krebs buffer and equipment calibration, following anesthetization with isoflurane (5%, Aerrane, Baxter Healthcare Corporation, Deerfield, IL, USA), rats (*n* = 22) were decapitated and colonic tissue was isolated and immediately immersed in ice cold oxygenated Krebs buffer containing (mM): 121 NaCl, 5.95 KCl, 1.19 MgCl_2_, 2.50 CaCl_2_, 1.34 NaH_2_PO4, 14.3 NaHCO_3_, 11.4 C_6_H_12_O_6_. Colonic tissue was opened along the mesenteric border and 1.5 cm segments of mucosa stripped of myenteric plexus and longitudinal muscular layers were mounted in four modified Ussing chambers (W-P Instruments Inc., Sarasota, FL, USA) with an aperture of 0.6 cm^2^. Exposed surfaces of the epithelial segments were bathed in identical formulations of Krebs buffer, continuously perfused with 95% O_2_ and 5% CO_2_, while bath temperature was maintained at 37°C by a water heating circulator (Julabo, Allentown, PA, USA). All Krebs buffer chemicals were purchased from MilliporeSigma (St. Louis, MO, USA).

### Ussing chamber experiments for the measurement of TEER

Colonic epithelial segments were mounted in Ussing chambers and equilibrated in Krebs buffer at 37°C for 30 min before potential difference (PD) and short-circuit current (Isc) across the epithelial segment were monitored. If the chamber did not demonstrate a stable baseline Isc after the 30 min equilibration period, the tissue was replaced with another segment from the same animal (up to two additional chambers were attempted) and the new chamber received an additional 30 min equilibration period. No electrophysiological measurements were conducted if a stable Isc could not be recorded. Two voltage sensing, agar-salt bridge electrodes on either side of the tissue monitored the PD across the tissue, while two Ag-AgCl current passing electrodes injected current into the system to maintain a PD of zero. PD and Isc were measured using an EVC 4000 Precision V/I clamp (W-P Instruments Inc., Sarasota, FL, USA). Stable baseline values for PD and Isc confirmed viability of the tissue preparation. Transepithelial electrical resistance (TEER) across the epithelial layer was calculated from Ohm's law using measures of PD and Isc and corrected for the area of the aperture normalized to 1 cm^2^:

$$\begin{array}{l} \text{TEER}\left(\text{Ω}.cm^{2}\right)=\left[\text{PD}\left(\text{mV}\right)/{\text{I}}_{\text{sc}}\left(\mu A\right)\right]\left(1/0.6\right) \end{array}$$

### Ussing chamber experiments for the measurement of macromolecular transport

Macromolecular transport was assessed by measuring horseradish peroxidase (HRP) type II (~44 kDa), (MilliporeSigma, St. Louis, MO, USA) flux from the mucosal to the serosal sides of the colonic epithelial layer. HRP in Krebs solution (0.8 mg/mL) was added to the mucosal bath of the Ussing chamber, and samples (0.25 mL) were taken from the submucosal bath at 20 min intervals for 2-h following equilibration. An equivalent volume of fresh Krebs buffer was added to the serosal bath to maintain equal hydrostatic pressure on either side of the mounted tissue. A colorimetric endpoint assay was used to quantify the amount of HRP contained in each sample. 3,3′,5,5′-tetramethylbenzidine (TMB) liquid substrate (25 μl) (MilliporeSigma, St. Louis, MO, USA) was incubated at room temperature with the HRP containing sample (1 μl) for 10 min followed by the addition of 0.1M H_2_SO_4_ (25 μl) to stop the HRP-TMB reaction. Samples were read at 450 nm in a Dynex MRX colorimetric plate reader (Magellan Biosciences, Chantilly, VA, USA), using Revelation plate reading software (Chantilly, VA, USA). The concentration of HRP present in each sample was determined using a standard curve. During the 2-h sampling period six samples were collected for HRP analysis and the average HRP flux was expressed as ngHRP/hr/cm^2^.

### Ussing chamber experiments for the measurement of active transport-induced by neural stimulation

To assess whether elevated amygdala CORT affected net active ion transport, electrical field stimulation (EFS) was used to induce nerve mediated increased in short-circuit current (Isc). With an electromotor force of 100 mV, electrical current (pulse duration: 0.5 ms; frequencies 1-64 Hz; 1 s train duration) was passed through two foil electrodes situated on the serosal side of the mounted colon segment, connected to a Grass S88 Stimulator using a Grass SIU5 stimulus isolation unit (Grass Instrument Co., Quincy, MA, USA). The maximum increase in Isc was compared with pre-stimulation baseline Isc to quantify the post-stimulation response at each protocol frequency. EFS data acquired using PowerLab 8 data acquisition system and LabChart7 Software (AD Instruments, Colorado Springs, CO, USA). In a sub-set of tissues, forskolin (10 μM) was added bilaterally to the mucosal and serosal sides of the Ussing chamber. Forskolin is known to increase intracellular cAMP production and induce an increase in Isc across the epithelial cell layer (Nemeth et al., 1986).

### Tissue isolation for histological studies

Within the main laboratory, starting at 10:00 a.m., following anesthetization with isoflurane (5%, Aerrane, Baxter Healthcare Corporation, Deerfield, IL, USA), rats (*n* = 10) were decapitated and the colon tissue was isolated, opened along the mesenteric border, and immediately immersed in ice cold oxygenated Krebs buffer. 1.5 cm colonic segments were placed in 10% neutral buffered formalin at room temperature for histological evaluation.

### Colonic histology

Formalin fixed tissue was processed in xylene and successive treatments of EtOH (95 and 100%). Tissue segments were paraffin embedded and sliced at 5 μm using a rotary microtome and slides were cured at 37°C. Slides were deparaffinized in xylene (2 × 10 min) and successive treatments of EtOH (100% × 2 × 3 min; 95 × 2 × 3 min; 70% × 3 min) and then rehydrated in diH2O. Slides were stained with commercially available Hematoxylin solution (StatLab, McKinney, TX) followed by Eosin counterstaining (StatLab, McKinney, TX) followed by dehydration in EtOH (95, 95, 100, 100%). Tissues were visualized using a light microscope and scored for histological damage by an expert pathologist using the Chui score for mucosal damage (Chiu et al., 1970).

### Mast cell stain

Colonic epithelial segments were evaluated for mast cell counts across all treatment and control groups. Slides were deparaffinized in successive treatments of xylene and EtOH as described above (see Hematoxylin/Eosin Staining) and stained using the standard toluidine blue mast cell staining procedure. Toluidine blue stock solution was prepared using 1 g toluidine blue-O (MilliporeSigma, St. Louis, MO) in 100 ml 70% EtOH. NaCl stock solution (1%) was prepared with 0.5 g NaCl:50 ml diH_2_O. The working toluidine blue solution consisted of 5 ml toluidine blue stock solution: 45 ml 1% NaCl solution and the pH was adjusted with glacial acetic acid for optimal staining conditions (pH = 2.5). Slides were immersed in the working solution for 1 min then rinsed in diH_2_O 3 times. Slides were dehydrated in 95 and 100% EtOH (3 rapid immersions each) and cleared by 2 × 3 min in xylene. Tissue was visualized by light microscopy. Mast cell counts were quantified by a blinded observer by counting the number of mast cells visible in 10 high powered fields at 100 × magnification from the distal colon of 4 rats/treatment, with the final result expressed as the average number of mast cells per μm^2^.

### Statistical analysis and experimental rigor

All data, expressed as mean ± SEM, was assessed for normality using the Shapiro-Wilk normality test before testing for statistically significant differences between the treatment and control groups (CORT vs. CHOL; ^*^p < 0.05, ^**^p < 0.01) using an unpaired Student's *t*-test for parametric data or a Mann-Whitney test for nonparametric data. Results for TEER, I_sc_, macromolecular flux of HRP, and mast cell counts were evaluated in this manner. Data from electrical field stimulation were evaluated using a repeated measure 2-factor analysis of variance (ANOVA), with Bonferroni *post-hoc* testing (^**^p < 0.01). Graphpad Prism 7 (Graphpad Software, La Jolla, CA, USA) was used for all analysis. Although the experimenter was not blinded to the experimental treatments, the histologist was blinded. While a power analysis was not conducted, *n*-values for this study were similar to our previously published reports using the same techniques (Venkova et al., 2004; Venkova and Greenwood-van Meerveld, 2006; Tran and Greenwood-Van Meerveld, 2013; Hattay et al., 2017). For the I_sc_ and TEER calculations, individual chambers were excluded when the PD was >−10 mV or < −1 mV—indicating either an error with the electrodes or a damaged tissue preparation; when excluding chambers due to PD measurement, the I_sc_, calculated TEER, and the HRP flux for that chamber were also excluded from analysis.

## Results

### Elevated amygdala cort causes abnormal colonic macromolecular flux

Seven days after amygdala implant surgery with CORT or CHOL containing micropellets, colonic tissue was isolated and Isc assessed *ex-vivo* in modified Ussing chamber experiments. Our results showed there was no significant difference (*t* = 0.357, df = 28, *p* = 0.724) in baseline Isc across the distal colonic epithelium of CORT (31.78 ± 3.0 μA/cm^2^) and CHOL (33.31 ± 3.1 μA/cm^2^) implanted rats (*n* = 5–6 rats, 14–16 chambers/treatment). In the same experimental preparation, we measured TEER and found that there was no significant change in TEER (Mann–Whitney *U* = 88.5, *p* = 0.339) across the colonic epithelium from CORT implanted rats compared to tissue from CHOL implanted controls (Figure 1C). As illustrated in Figure 1D, the macromolecular flux of HRP was significantly increased (Mann–Whitney *U* = 16, *p* = 0.0024) across the colonic epithelium of rats with amygdala CORT implants (*n* = 4 rats, 11 chambers) compared to amygdala CHOL control animals (*n* = 5 rats, 11 chambers). In all animals, we verified the correct localization CORT or CHOL micropellet on the dorsal margin of the CeA (Figure 1B).

### Elevated amygdala cort inhibits the nerve-mediated increase in Isc without affecting forskolin-induced Isc

**Figure 2:**
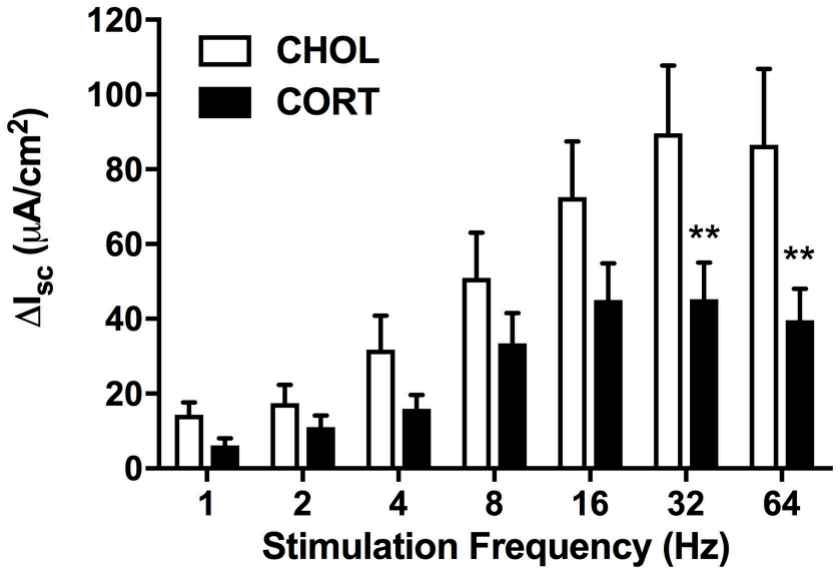
Active transport following electrical field stimulation is significantly inhibited in CORT implanted animals. Following EFS at frequencies of 32 and 64 Hz colonic tissue from CORT treated animals exhibited an attenuated Isc (Bonferroni *post-hoc* test, **p < 0.01) compared to CHOL treated controls. Values are expressed as mean ΔI_sc_ ± SEM following each stimulation pulse. Analyzed using 2-factor repeated measures ANOVA. (CHOL *n* = 4 rats, 8 chambers; CORT *n* = 5 rats, 14 chambers).

**Figure 3:**
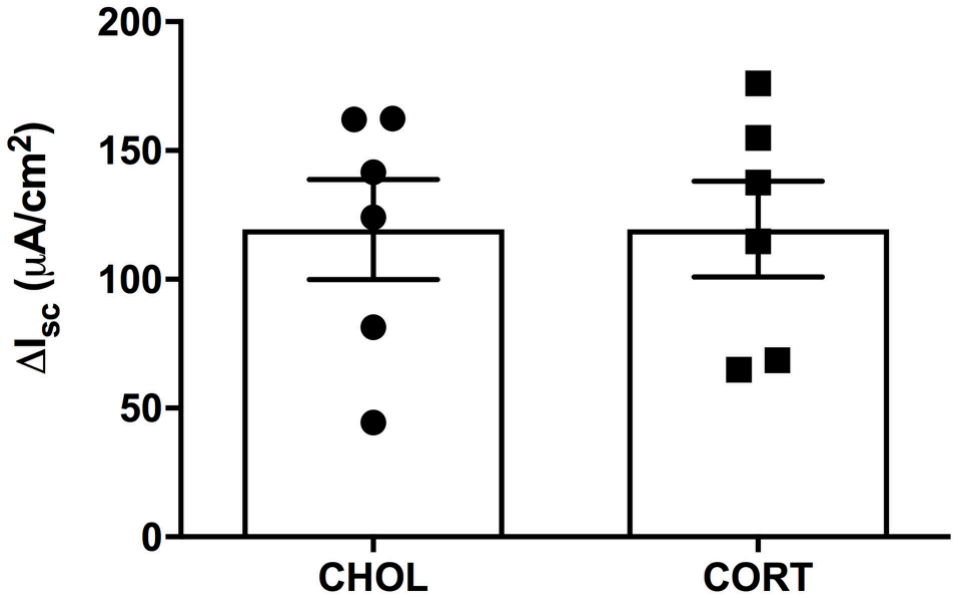
CORT and CHOL treated animals exhibit similar cellular responses to increased cAMP induced by forskolin. The cellular response to mucosal and serosal administration of forskolin (10 μM) to the baths is not significantly different between CORT and CHOL implanted animals (*t* = 0.005, df = 10, *p* = 0.996, *n* = 2 rats, 6 chambers/treatment). Values are expressed as mean ΔI_sc_ ± SEM.

Here we aimed to investigate whether elevated amygdala CORT had any effect on active ion transport induced by stimulation of enteric nerves with EFS. In colonic tissue isolated from rats with CHOL implants on the dorsal margin of the CeA we found that EFS caused a frequency-dependent increase in Isc (Figure 2). Overall, there was a significant effect of EFS frequency [*F*_(6, 120)_ = 37.75, *p* < 0.0001], a significant effect of micropellet implant [*F*_(1, 20)_ = 6.763, *p* = 0.017], and a significant interaction between the terms [*F*_(6, 120)_ = 7.479, *p* < 0.0001] as demonstrated in colonic tissue isolated from CORT implanted rats (*n* = 5, 14 chambers) where there was a blunted Isc response to EFS at frequencies of 32 and 64 Hz compared to the Isc responses in colonic tissue from CHOL controls (*n* = 4, 8 chambers). In a separate cohort of experiments, we investigated whether forskolin (10 μM)-induced Isc was effected by elevated amygdala CORT. Addition of forskolin caused a rapid and significant increase in Isc, however there was no difference in the magnitude of the forskolin-induced increase in Isc (*t* = 0.005, df = 10, *p* = 0.996) between tissue isolated from rats with CORT or CHOL micropellets implanted on the dorsal margin of the CeA (*n* = 2 rats, 6 chambers/treatment) (Figure 3).

### Elevated amygdala cort causes no changes in colonic histology or mast cell infiltration

**Figure 4:**
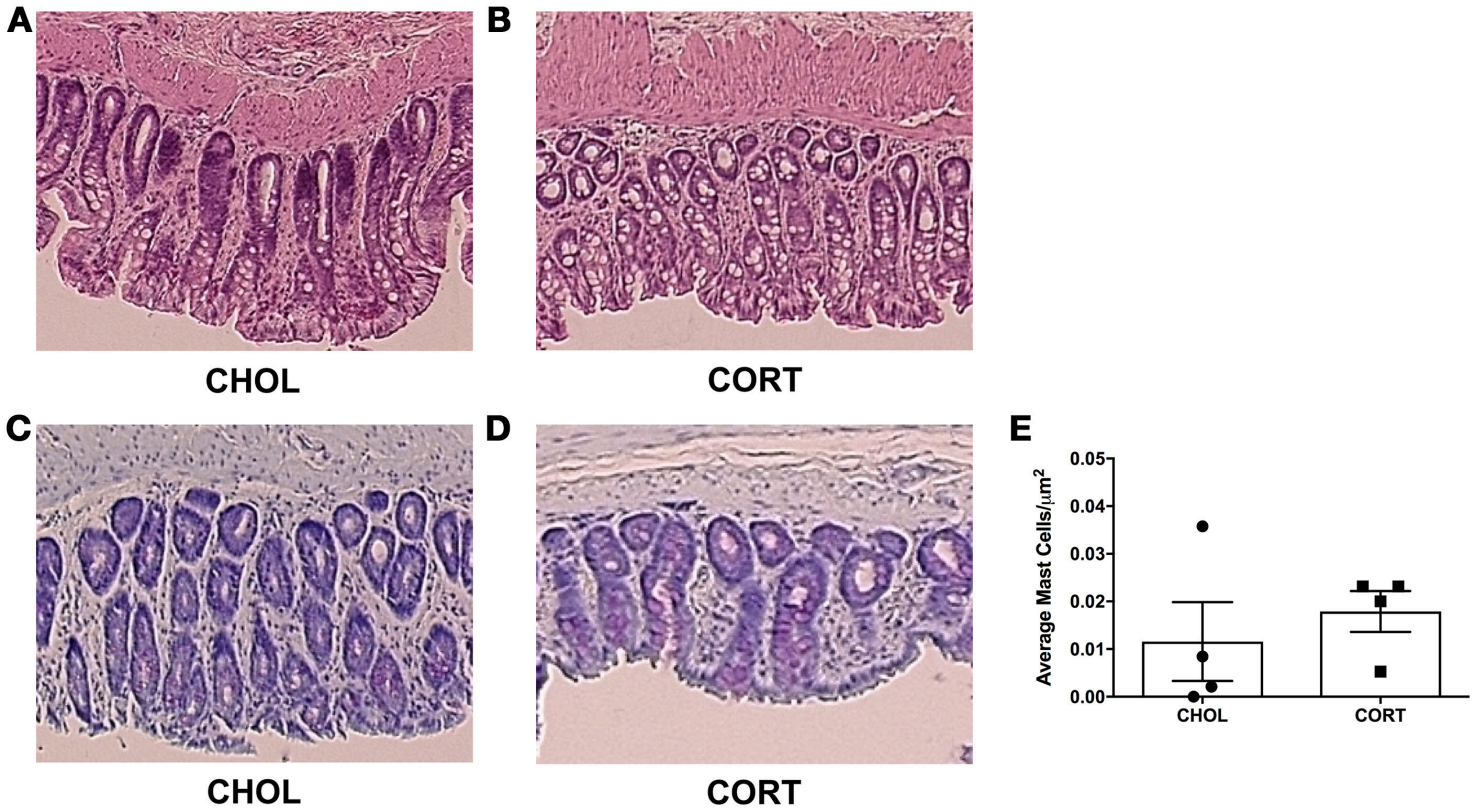
Histological appearance and mast cell infiltration following CORT or CHOL amygdala implants onto the CeA. **(A,B)** Representative H&E stained colon section. A score of zero was given across to all specimens across both treatment groups, indicating no mucosal damage in CORT or CHOL amygdala implanted animals. **(C–E)** Representative toluidine blue stained colon section. Mast cell infiltration was not increased following exposure of the CeA to CORT compared to CHOL treated controls (Mann–Whitney *U* = 5, *p* = 0.429, *n* = 4/treatment). Values are mean ± SEM.

A pathologist blinded to the treatment groups evaluated tissue samples stained with hematoxylin and eosin using a validated scoring system (Chiu et al., 1970). A score of zero was given to all specimens across both treatment groups, indicating neither CHOL control implantation nor CORT applied to the CeA induce histological changes within the colonic mucosa (Figures 4A,B). Colonic tissue samples were also stained to localize and quantify the number of mast cells. We found no differences in mast cell infiltration between CORT and CHOL treated animals (Mann–Whitney *U* = 5, *p* = 0.429, *n* = 4/treatment) (Figures 4C–E).

## Discussion

The present study shows that in an experimental model of central stress dysregulation, bilateral micropellets of CORT placed stereotaxically onto the dorsal margin of the amygdala, produced significant changes in colonic epithelial transport compared to CHOL-implanted controls in the absence of any histological changes within the colon. Through an *ex-vivo* assessment of colonic permeability via measurements of TEER, we found no changes in electrical resistance across the tissue of CORT-treated rats compared to CHOL controls. Abnormalities in colonic barrier function induced by direct pharmacological activation of the CeA were supported by the results showing that amygdala CORT-treated rats exhibited enhanced macromolecular flux across the colonic mucosa compared to CHOL controls. In contrast, active ion transport across the colonic mucosa induced by electrical stimulation was significantly inhibited in tissue isolated from rats in which the CeA was activated with a CORT-containing micropellet without changes in cAMP-induced active ion transport. Further, there were no overt changes in histological appearance or mast cell infiltration of the colonic mucosa. The data from the current study suggests that activation of the elevated amygdala CORT, has profound effects on the regulation of colonic barrier function and active ion transport in the absence of marked changes to the arrangement of the colonic epithelia.

IBS is characterized by chronic visceral pain and abnormal bowel habits that are worsened by stress. Previous studies have shown in experimental models and human volunteers that stress has profound effects on the mucosal barrier (Söderholm and Perdue, 2001; Vanuytsel et al., 2014), however, the mechanism by which stress increases colonic permeability is not well understood. Previously, we demonstrated in an experimental rat model that activation of the CeA with the stress hormone CORT induced persistent visceral hyperalgesia and anxiety-like behavior (Greenwood-Van Meerveld et al., 2001; Myers and Greenwood-Van Meerveld, 2007; Myers et al., 2007), however, the effect of CeA activation with CORT on colonic permeability remained to be evaluated. In the current study, using an experimental model in which we are able to isolate and activate only the CeA by stereotaxically placing micropellets of CORT directly onto the dorsal margin of the CeA, we found that elevated amygdala CORT had profound effects on colonic permeability. Specifically, when comparing the results of our current study in which we selectively activated the CeA to our previous study using a model of chronic psychological stress (Hattay et al., 2017), both models demonstrated a significantly increased macromolecular flux across the colon. In contrast to the stress model, we found that elevated CeA CORT did not alter TEER or increase colonic mast cell infiltration. The mechanism(s) by which CeA CORT increases the colonic macromolecular flux of HRP without an effect on TEER across the isolated colonic mucosal remains incompletely understood but suggests an effect that is independent of a change in electrical resistance across the tissue. However, a similar phenomenon was observed in a model of traumatic brain injury in which the associated increase in dextran flux in the absence of a change in TEER was associated with a decrease in Claudin-1 mucosal expression (Ma et al., 2017). While not currently investigated in this study, data from IBS patient biopsies have demonstrated barrier dysfunction linked to down regulation in the expression and redistribution of the scaffolding tight junction (TJ) protein ZO-1, whereas levels of the TJ protein occludin were unchanged (Piche et al., 2009). Another study showed significantly increased staining of occludin in the cytoplasm of jejunal enterocytes, suggesting intensive internalization of this protein, compared to healthy controls where occludin was predominantly located in the TJ (Martinez et al., 2012). Future studies will investigate whether the increase in macromolecular flux-induced by the CORT micropellet on the CeA was due to changes in TJ expression.

Previous studies have also shown that colonic secretion driven predominantly by active chloride secretion is important for lubrication and hydration of the colonic mucosal surface. Here we investigated whether amygdala activation with CORT influences active ion transport induced by stimulation of enteric nerves via EFS. Acetylcholine (ACh) is the most predominant neurotransmitter in the ENS and in response to EFS the release of ACh stimulates chloride secretion from the colonic crypts measured electrophysiologically as an increase in Isc. In the current study, we found that in control CHOL implanted rats, EFS induced a frequency dependent increase in Isc; however, in rats with CORT-containing micropellets on the CeA there was a significant attenuation in the EFS-induced increase in Isc, which was in contrast to the lack of change in the EFS response following a chronic psychological stressor (Hattay et al., 2017). These findings suggest that isolated exposure of the CeA to CORT alters nerve-mediated active ion transport in the colon, whereas other brain regions recruited during chronic psychological stress prevent the amygdala-induced effect on EFS. These results are in contrast to our findings that the receptor independent cAMP-mediated increase in Isc induced by forskolin is unaffected by CORT-induced amygdala activation or chronic stress exposure (Hattay et al., 2017). These results support the notion that long-term exposure of the CeA to elevated levels of the stress hormone CORT is capable of specifically inhibiting nerve-mediated active secretion in the colon. However, future experiments are required to determine the physiological relevance of this reduction in EFS-induced secretion in response to elevated amygdala CORT and must be reconciled with the concurrent increase in colonic macromolecular flux seen in rats exposed to chronic activation of the CeA with CORT.

Important to the interpretation of our latest results is that changes in colonic epithelial transport occurred in the absence of changes in colonic histology. These data mirror IBS patient pathology where there is no obvious tissue damage to explain patient symptomatology including visceral pain and abnormal bowel habits (Kirsch and Riddell, 2006; Enck et al., 2016). Several luminal factors have been linked to increases in gut permeability including diet, food allergies, and changes in the gut microbiota (Camilleri et al., 2012; Bischoff et al., 2014). Recent evidence from patients with IBS suggests that there is immune system activation with increases in mast cell numbers and mast cell degranulation (Barbara et al., 2011; Martinez et al., 2012). Interestingly, in our experimental model of CeA activation we did not observe any differences in the number of mast cells within the colonic mucosa between rats implanted with CORT or CHOL on the CeA. However, in two separate models of chronic stress, an increase in the number of degranulated mast cells (Santos et al., 2001), and an overall increase in total mast cells within the colonic mucosa (Hattay et al., 2017) have been observed. Previous studies have indicated that mast cell mediators have the potential to alter tight junction formation and diminish barrier integrity (Piche, 2014; Lee and Lee, 2016). While we did not see an increase in mast cell infiltration, it is possible that tight junction proteins may have changed within the CORT treated animals due to differential release of mast cell mediators, and not simply due to increased infiltration of mast cells into the colonic epithelium. For this reason, future studies will be aimed at comparing mast cell mediator release, such as tryptase between amygdala CORT and CHOL treated rats.

Our study was not designed to mimic physiological concentrations of CORT in response to stress, rather an advantage of our experimental approach is that we are able to investigate the effect of amygdala-specific CORT activation on the integrity of colonic barrier function. The concentration of CORT was chosen based on our previous observations showing increases in colonic hypersensitivity in rats without spread of the CORT to other structures in the region of the amygdala (Myers and Greenwood-Van Meerveld, 2007). In the current series of experiments, CORT implants were placed on the dorsal margin of the amygdala to ensure that CORT bathed the CeA without any physical damage to the structure (Myers and Greenwood-Van Meerveld, 2010a). The concentration of CORT in tissue micropunches taken from sites surrounding the CORT micropellet indicated a diffusion radius of ~0.5–1 mm which would include the CeA but exclude other central sites expressing corticosteroid receptors such as the hippocampus, paraventricular nucleus of the hypothalamus and bed nucleus of the stria terminalis (BNST) (Shepard et al., 2003). Finally, in a previous study by targeting the hippocampus and caudate putamen with CORT micropellets we observed levels of colonic sensitivity and anxiety that were similar to cholesterol controls verifying that the effects of CORT on anxiety and colonic hypersensitivity were amygdala specific (Myers and Greenwood-Van Meerveld, 2007).

Taken together our findings support a role for the CeA as a key modulator of colonic epithelial function that may play a role in producing the previously observed increase in visceral hypersensitivity seen in response to elevated amygdala CORT (Greenwood-Van Meerveld et al., 2001; Myers and Greenwood-Van Meerveld, 2007; Myers et al., 2007). While the exact pathway by which elevated amygdala CORT induces colonic epithelial dysfunction remains to be determined, our findings confirm that activation of the CeA with the stress hormone CORT can produce dysfunction within the colonic epithelium. Projections from the CeA that communicate with the gut through the autonomic nervous system via the periaqueductal gray, dorsal motor nucleus of the vagus, and the raphe nuclei, allow far reaching effects of the CeA on the periphery (Neugebauer et al., 2004). The amygdala interacts with the HPA axis through multisynaptic pathways connecting to the hypothalamic nuclei (lateral, ventromedial, and periventricular) and the BNST (Dent et al., 2007). Therefore, there are potential neuronal pathways for peripheral effects that may be responsible for previously noted changes in visceral nociception, as well as alterations in epithelial barrier function that are centrally driven from the CeA.

In summary, many studies have examined various aspects of stress-induced symptom exacerbation as it pertains to colonic barrier dysfunction in animal models of IBS. To our knowledge, we are the first focus on the role of the amygdala, specifically the CeA, in altering colonic barrier function and nerve-mediated active ion transport. Our findings support a role for the amygdala as a key central modulator of colonic epithelial function and integrity as it relates to gut health and the pathophysiology of functional bowel disorders such as IBS.

## Author contributions

PH and BG-VM conceptualized the experiments. PH and DP performed the experiments. PH, DP, and AJ analyzed the results. PH and BG-VM wrote the first draft of the manuscript. PH, DP, AJ, and BG-VM revised the manuscript.

### Conflict of interest statement

The authors declare that the research was conducted in the absence of any commercial or financial relationships that could be construed as a potential conflict of interest.

## Abbreviations

ACh: acetylcholine
ANOVA: analysis of variance
BNST: bed nucleus of the stria terminalis
CeA: central nucleus of the amygdala
CHOL: cholesterol
CORT: corticosterone
diH_2_O, deionized: distilled water
EFS: electrical field stimulation
F-344: Fischer 344 rat
FBDSI: functional bowel disorder severity index
GI: gastrointestinal
HPA: hypothalamic-pituitary-adrenal axis
HRP: horseradish peroxidase
IBS: irritable bowel syndrome
Isc: short-circuit current
PD: potential difference
SEM: standard error of the mean
TEER: transepithelial electrical resistance
TJ: tight junction
TMB, 3, 3′, 5: 5′-tetramethylbenzidine
ZO: zonula occludens.
